# The mouse fibula as a suitable bone for the study of functional adaptation to mechanical loading

**DOI:** 10.1016/j.bone.2008.12.026

**Published:** 2009-05

**Authors:** Alaa Moustafa, Toshihiro Sugiyama, Leanne K. Saxon, Gul Zaman, Andrew Sunters, Victoria J. Armstrong, Behzad Javaheri, Lance E. Lanyon, Joanna S. Price

**Affiliations:** Department of Veterinary Basic Sciences, The Royal Veterinary College, University of London, Royal College Street, London NW1 0TU, UK

**Keywords:** Mechanical loading, Functional adaptation, Fibula, Parathyroid hormone, Sclerostin

## Abstract

Bones' functionally adaptive responses to mechanical loading can usefully be studied in the tibia by the application of loads between the knee and ankle in normal and genetically modified mice. Such loading also deforms the fibula. Our present study was designed to ascertain whether the fibula adapts to loading in a similar way to the tibia and could thus provide an additional bone in which to study functional adaptation. The right tibiae/fibulae in C57BL/6 mice were subjected to a single period of axial loading (40 cycles at 10 Hz with 10-second intervals between each cycle; approximately 7 min/day, 3 alternate days/week, 2 weeks). The left tibiae/fibulae were used as non-loaded, internal controls. Both left and right fibulae and tibiae were analyzed by micro-computed tomography at the levels of the mid-shaft of the fibula and 25% from its proximal and distal ends. We also investigated the effects of intermittent parathyroid hormone (iPTH) on the (re)modelling response to 2-weeks of loading and the effect of 2-consecutive days of loading on osteocytes' sclerostin expression. These *in vivo* experiments confirmed that the fibula showed similar loading-related (re)modelling responses to those previously documented in the tibia and similar synergistic increases in osteogenesis between loading and iPTH. The numbers of sclerostin-positive osteocytes at the proximal and middle fibulae were markedly decreased by loading. Collectively, these data suggest that the mouse fibula, as well as the tibia and ulna, is a useful bone in which to assess bone cells' early responses to mechanical loading and the adaptive (re)modelling that this engenders.

## Introduction

It is generally accepted that a number of the most important features of bone architecture, particularly those on which its load-bearing competence depends, are only achieved and maintained as a result of an adaptive response by resident bone cells to load-induced strain in their matrix [1]. Investigation of the objectives of strain-related (re)modelling and the mechanisms involved requires availability of bones that can be loaded *in vivo* in experimental animals. Early models in sheep [2], turkeys [3], roosters [4] and rats [5–7] have been followed by those in mice [8–12]. Using normal and genetically modified mice, the non-invasive axial loading model of the ulna has proved useful in a number of experiments to study cortical bone [9,13–17], as has the recently introduced, non-invasive axial loading model of the tibia [11,12,17–21]. The latter model has the advantage of enabling the study of trabecular as well as cortical compartments.

Since the fibula is attached to the tibia, both bones are loaded when mechanical loads are axially applied between the knee and ankle. In this article, we report the adaptive (re)modelling responses of the mouse fibula to axial loading alone and in conjunction with intermittent parathyroid hormone (iPTH) (1–34). We also report the effect of this loading on sclerostin expression in the fibula's osteocytes.

## Materials and methods

### Animals

Virgin, female C57BL/6 mice at 7–8 weeks of age were purchased from Charles River Laboratories, Inc. (Margate, UK) and group-housed in sterilized polypropylene cages with free access to water and a maintenance diet containing 0.73% calcium, 0.52% phosphorus, and 3.5 IU/g vitamin D (RM1; Special Diet Services Ltd., Witham, UK) in a 12-hour light/dark cycle, with room temperature at 21 ± 2 °C. All procedures complied with the UK Animals (Scientific Procedures) Act 1986 and were reviewed and approved by the ethics committee of the Royal Veterinary College (London, UK).

### *In vivo* external mechanical loading

The apparatus and protocol for dynamically loading the mouse tibia/fibula have been reported previously [11,17,18,22]. Dynamic axial loads (0.1 s trapezoidal-shaped pulse period [0.025 s loading, 0.05 s hold and 0.025 s unloading]; 10 s rest time between pulses; 40 cycles/day) were applied between the right flexed knee and ankle under isoflurane-induced anesthesia (approximately 7 min/day). In brief, the flexed joints are positioned in concave cups; the upper cup, into which the knee is positioned, is attached to the actuator arm of a servo-hydraulic loading machine (Model HC10; Zwick Testing Machines Ltd., Leominster, UK) and the lower cup to a dynamic load cell. The servo-hydraulic mechanism of the loading machine operates to apply controlled dynamic compressive loads axially to the tibia/fibula. The left tibia/fibula was used as a non-loaded, internal control. Normal cage activity was allowed between loading periods.

### ‘Loading’ experiment

When the mice were 19 weeks of age, their right tibiae/fibulae were subjected to single short periods of loading on 3 alternate days per week for 2 weeks. Strain gauges attached to the medial surface of the tibial shaft showed that a peak load of 13.5 N engendered approximately 1400 microstrain (μɛ) at a site 37% distal to its proximal end. Unfortunately the fibula was too small to allow the attachment of currently available strain gauges to its surface.

**Fig. 1:**
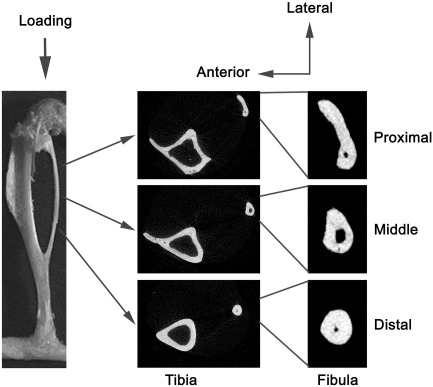
Direction of mechanical loading in the right tibia/fibula and representative transverse μCT images of the fibula and tibia at the analyzed sites (25% proximal, middle and 25% distal sites of the fibula's length) in a 19 week old female C57BL/6 mouse.

Calcein (30 mg/kg; Sigma Chemical Co., St. Louis, Missouri, USA) was injected intraperitoneally on the first and last days of loading (days 1 and 12). The mice were killed at day 15, and their tibiae and fibulae were collected and stored in 70% ethanol before being scanned with micro-computed tomography (μCT) with a pixel size of 5 μm (SkyScan 1172; SkyScan, Kontich, Belgium). The images of the whole bones were reconstructed by the SkyScan software and their lengths were measured. As shown in Fig. 1, the fibulae and tibiae were analyzed at the same three sites, i.e., 0.5 mm long sections at proximal (25%), middle (50%) and distal (25%) sites along the fibula's length. Periosteally-enclosed volume, cortical bone volume and medullary volume were measured. After scanning by μCT, the bones were dehydrated, cleared and embedded in methyl methacrylate as previously described [23]. Transverse segments of the fibulae were obtained by cutting with an annular diamond saw. Images of calcein labels were visualized using the argon 488 nm laser of a confocal laser scanning microscope (LSM 510; Carl Zeiss MicroImaging GmbH, Jena, Germany) at similar regions as the μCT analysis.

### ‘iPTH’ experiment

Mice in this part of the study, starting when they were 13 weeks old, were given 6-weeks of intermittent treatment with subcutaneous human PTH (1–34) (iPTH; Bachem Biosciences, Inc., King of Prussia, Pennsylvania, USA) at a dose of 80 μg/kg/day (7 days/week) or vehicle (99.7% saline, 0.2% bovine serum albumin [Sigma Chemical Co.], and 0.1% hydrochloric acid). During the last 2 weeks (3 alternate days/week), the right tibiae/fibulae of these mice were subjected to loading between 30 and 40 min after the vehicle or PTH (1–34) injection. Since the mice receiving iPTH (1–34) had deposited more new bone than those which had not, it was necessary to apply different magnitudes of a peak load to achieve a similar level of strain. Thus, the mice treated with the vehicle or iPTH (1–34) received a peak load of 12.0 or 15.8 N, respectively. In each case, these peak loads engendered approximately 1200 μɛ at the medial surface of the tibiae 37% along the bone shaft from their proximal end, as previously reported [17]. This level of strain was designed to be lower than that (approximately 1400 μɛ) in the ‘loading’ experiment, to provide scope for observing the synergistic osteogenic effect with iPTH (1–34) that we have reported previously [17].

Calcein (30 mg/kg; Sigma Chemical Co.) was injected intraperitoneally on the first days of iPTH (1–34) treatment (day 1) and loading (day 29), and alizarin (30 mg/kg; Sigma Chemical Co.) on the last day of loading (day 41). At 19 weeks of age (day 43), the animals were killed and their tibiae and fibulae collected. As described above, these bones were analyzed by μCT and the distribution of calcein and alizarin labels visualized by confocal laser scanning microscopy using argon 488 nm laser and HeNe 543 nm laser, respectively.

### ‘Sclerostin’ experiment

Mice in this experiment were subjected to loading with a peak load of 13.5 N on two consecutive days at 19 weeks of age. The fibulae on each side were collected 24 h after the second period of loading. These bones were dissected of soft tissue and fixed in 10% formalin. Sclerostin was immunolocalized at the proximal and middle sites in decalcified, wax-embedded 8-μm transverse sections using an indirect immunoperoxidase method [24]. Goat polyclonal anti-mouse sclerostin (0.2 mg/ml; R&D Systems, Abingdon, UK) and biotinylated rabbit anti-goat (0.013 mg/ml; Dako, Ely, UK) were used as the primary and secondary antibodies, respectively. All antibodies were diluted in 10% rabbit serum (Sigma Chemical Co.) in calcium and magnesium-free phosphate buffered saline (Gibco, Paisley, UK). The same concentration of goat IgG was substituted for the primary antibody to provide a negative control. Detection of sclerostin was carried out using vector ABC kit (Vector Laboratories, Burlingame, USA) with diaminobenzidine as a substrate. The immunolabeled sections were photographed using a Leica Q550IW light microscope (Leica Microsystems, Heidelberg, Germany). The numbers of sclerostin-positive and total osteocytes were counted and the ratio of sclerostin-positive to total osteocyte number was evaluated in two sections at each of the three sites. Using this ratio, mechanical load-induced percentage changes in osteocytes' sclerostin expression were then calculated ([right loaded − left control] × 100 / left control).

### Statistical analysis

All data are shown as mean ± S.E. Statistical analysis was performed by paired *t*-test, two-way ANOVA or one-way ANOVA followed by a post hoc Bonferroni or Dunnett T3 test using SPSS for Windows (version 16.0; SPSS Inc., Chicago, USA). *p* < 0.05 was considered as statistically significant.

## Results

### Effects of mechanical loading on the fibula

**Fig. 2:**
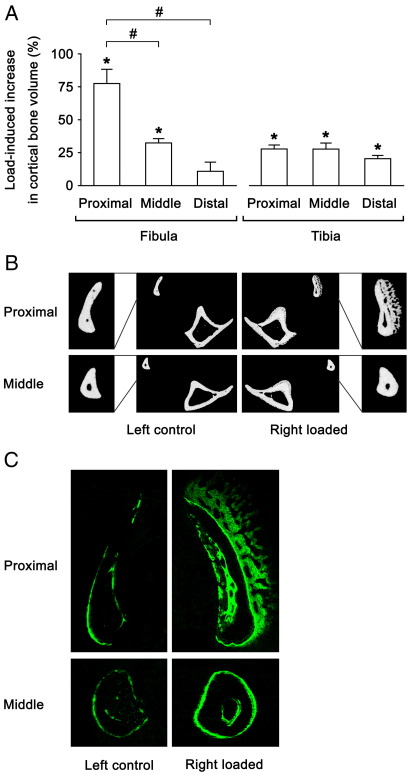
Effects of 2-weeks of mechanical loading with a peak load of 13.5 N on the fibula and tibia in 19 week old female C57BL/6 mice. A: Load-induced percentage increases ([right loaded − left control] × 100 / left control) in cortical bone volume of the fibula and tibia. Mean ± S.E. (*n* = 5). ⁎*p* < 0.05 by paired *t*-test between left control and right loaded. ^#^*p* < 0.05 by one-way ANOVA followed by a post hoc Bonferroni or Dunnett T3 test among different sites. B: Representative transverse μCT images of the fibula and tibia. C: Representative transverse fluorochrome labelled images of the fibula. Green: calcein labels injected on the first and last days of loading (days 1 and 12).

Table 1 and Fig. 2 show the results in the ‘loading’ experiment. Loading with a peak load of 13.5 N significantly increased the fibula's cortical bone volume at the proximal and middle sites (78 ± 11 % [*p* < 0.01] and 32 ± 3 % [*p* < 0.01], respectively). Only the middle site of the fibula included sufficient medullary volume for its inclusion in the analysis. At this level, loading significantly increased periosteally-enclosed volume (26 ± 3 % [*p* < 0.01]) and tended to decrease medullary volume (− 24 ± 13 % [*p* = 0.19]). Transverse μCT and fluorochrome labelled images in the fibulae showed that the character of new bone formed in response to loading was both lamellar and woven at the proximal site and lamellar only at the middle site.

### Effects of mechanical loading plus iPTH on the fibula

**Fig. 3:**
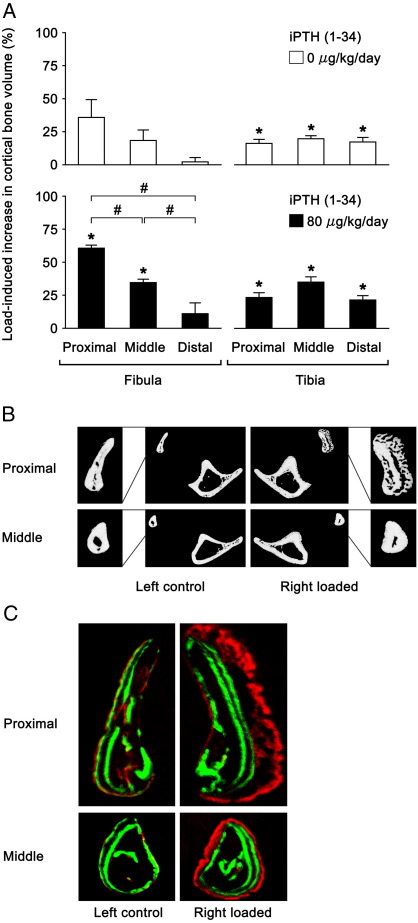
Effects of 2-weeks of mechanical loading with a peak load of 12.0 or 15.8 N, which produced a similar level of strain measured at the medial surface of the tibia, on the fibula and tibia in 17 week old female C57BL/6 mice treated with vehicle or iPTH (1–34) for 6 weeks, respectively. A: Load-induced percentage increases ([right loaded − left control] × 100 / left control) in cortical bone volume of the fibula and tibia. Mean ± S.E. (*n* = 5). ⁎*p* < 0.05 by paired *t*-test between left control and right loaded. ^#^*p* < 0.05 by one-way ANOVA followed by a post hoc Bonferroni or Dunnett T3 test among different sites. B: Representative transverse μCT images of the fibula and tibia in the mice treated with iPTH (1–34). C: Representative transverse fluorochrome labelled images of the fibula in the mice treated with iPTH (1–34). Green: calcein labels injected on the first days of iPTH (1–34) treatment (day 1) and loading (day 29). Red: alizarin label injected on the last day of loading (day 41).

The results in the ‘iPTH’ experiment are shown in Table 2 and Fig. 3. In the fibulae of mice treated with vehicle, loading with a peak load of 12.0 N also increased cortical bone volume at the proximal and middle sites, but these increases were not statistically significant (36 ± 14 % [*p* = 0.06] and 18 ± 8 % [*p* = 0.08], respectively). Treatment with iPTH (1–34) promoted these loading-related responses; loading increased cortical bone volume at the proximal and middle sites by 61 ± 2 % (*p* < 0.01) and 35 ± 3 % (*p* < 0.01), respectively. This represented a significant synergistic effect on cortical bone volume between loading and iPTH (1–34) at the proximal site (*p* = 0.01). Loading in combination with iPTH (1–34) treatment resulted in both lamellar and woven bone formation at the proximal site and only lamellar bone formation at the middle site.

### Effects of mechanical loading on osteocytes' sclerostin expression in the fibula

**Fig. 4:**
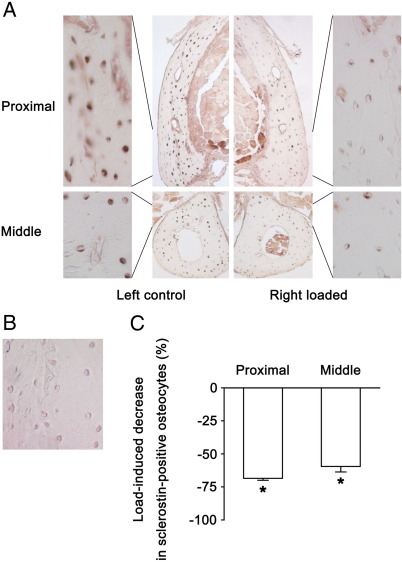
Effect of 2-consecutive days of mechanical loading with a peak load of 13.5 N on osteocytes' sclerostin expression in the fibula of 19 week old female C57BL/6 mice. A: Representative transverse sclerostin-immunostained images 24 h after the last loading. B: Negative control of sclerostin-immunostained image. C: Mechanical load-induced percentage decreases in sclerostin-positive osteocytes ([right loaded − left control] × 100 / left control). Mean ± S.E. (*n* = 6). ⁎*p* < 0.05 by paired *t*-test between left control and right loaded.

Twenty-four hours after the second period of loading, there was a reduction in the number of sclerostin-positive osteocytes at the proximal and middle sites of the fibulae (Table 3 and Fig. 4, A and B). The total number of osteocytes showed no change. The ratios of sclerostin-positive to total number of osteocytes were 0.74 ± 0.02 and 0.81 ± 0.04, respectively, in the left control and 0.23 ± 0.01 and 0.32 ± 0.02, respectively, in the right loaded bones. The level of load-induced decrease in sclerostin-positive osteocytes thus tended to be higher at the proximal site than at the middle site (Fig. 4C; − 69 ± 2 % and − 60 ± 4 %, respectively [*p* = 0.07]).

## Discussion

The results of this study show that the mouse fibula responds in a similar way to that expected from other long bones such as the ulna and tibia [16,17] in three different respects: adaptive bone (re)modelling to a short period of intermittent loading; synergism of that response with iPTH (1–34); and loading-related reduction in osteocyte sclerostin production. This indicates that the fibula as well as the ulna and tibia is appropriate for studies of functional adaptation to mechanical loading.

Similarly to the ulna [9] and tibia [11,12], the fibula showed site-specific differences in response to the axial loading. This trend was not changed by different magnitudes of peak load or iPTH (1–34) treatment. The shape of the skeleton is controlled by its mechanical environment, and the differences among bone sites in response to any one loading configuration will be determined *inter alia* by how different this loading environment is from that to which the site in question is habituated.

The ‘iPTH’ experiment was designed to assess whether iPTH (1–34) treatment sensitizes bone cells to mechanical stimulation in the fibula, as we have previously shown in the tibia [17]. The results in the ‘iPTH’ experiment induced by loading, which produced a lower level of strain (approximately 1200 μɛ), in combination with high-dose iPTH (1–34) treatment, were similar to those in the ‘loading’ experiment which involved a higher level of strain (approximately 1400 μɛ) alone. These data, in addition to our previous findings in the tibia and ulna [17], support the hypothesis that iPTH reduces the peak load necessary to stimulate an anabolic effect in cortical bone.

It has been shown that iPTH inhibits SOST transcription *in vivo* [25]. A recent study using the axial loading model of the ulna [16] showed that in cortical bone osteocytes' expression of sclerostin, the protein product of the SOST gene, is regulated by mechanical strain. At the distal ulna where this loading induced a substantial increase in bone formation, the number of sclerostin-positive osteocytes were markedly decreased (by approximately 60%). Consistent with these findings, in the present study, mechanical loading with a peak load of 13.5 N induced marked decreases in the numbers of sclerostin-positive osteocytes at the proximal (mean 69% reduction) and middle (mean 60% reduction) sites of the fibula's cortical bone.

One advantage of the mouse fibula is that its small size facilitates analysis by nano-CT, which can provide information concerning osteocytes [26], and microscopic examination since a single field can encompass the whole bone's cross section. On the other hand, this small size also presents a disadvantage in that it is impossible to attach currently available strain gauges to the bone's surfaces and thus the actual strain levels associated with any particular response need to be inferred from engineering analysis rather than assessed by direct measurement. Knowledge of the precise strain levels at each location of loaded bones is, however, not a feature of many studies on functional adaptation.

We and others have routinely assessed the effects of mechanical loading by applying loads to bones in one limb and comparing the resulting (re)modelling with that of the contra-lateral limb that has received no artificial load [8–21,27–29]. However, a recent study by Sample et al. [30] has thrown doubt on the previously accepted assumption that the adaptive effects of loading are confined to the bones that are loaded, and thus loading has no effect on the bones not subjected to load, including those commonly used as controls on the contra-lateral side. In Sample's experiments, loading of the rat ulna was associated with remote regional and systemic effects on (re)modelling that were modified by analgesia of the brachial plexus of the loaded limb [30]. These findings of remote perturbations of (re)modelling do not accord with our experience. However, should they be shown to be generally applicable it will no longer be possible to assume that adaptive (re)modelling is a local response to strain confined to the loaded bone. Our current study, and all of those by our laboratory and others, which have used non-loaded contra-lateral bones as controls [8–21,27–29], must therefore be viewed with this caveat until the general applicability of Sample's study [30] can be ascertained.

In conclusion, the mouse fibula appears to be a suitable bone in which to assess mechanically adaptive (re)modelling associated with artificial loading of the tibia/fibula *in vivo*. Its small size offers some advantages in relation to imaging which must be weighed against the disadvantage that it is too small to attach strain gauges and thus measure strain levels directly. Since axial loading of the tibia also engenders strains in the fibula, inclusion of the fibula in the analysis of these experiments would increase the usefulness of the non-invasive, dynamic axial loading model of the mouse tibia/fibula.

## Figures and Tables

**Table 1:** Structural parameters in the fibula of 21 week old female C57BL/6 mice which had received 2-weeks of mechanical loading with a peak load of 13.5 N

	Left control	Right loaded	*p* value^a^
*Proximal site*			
Cortical bone volume (mm^3^)	0.062 ± 0.002	0.109 ± 0.006	< 0.01

*Middle site*			
Cortical bone volume (mm^3^)	0.048 ± 0.001	0.064 ± 0.002	< 0.01
Periosteally-enclosed volume (mm^3^)	0.054 ± 0.002	0.068 ± 0.003	< 0.01
Medullary volume (mm^3^)	0.0057 ± 0.0007	0.0042 ± 0.0009	0.19

*Distal site*			
Cortical bone volume (mm^3^)	0.040 ± 0.001	0.044 ± 0.002	0.21

Mean ± S.E. (*n* = 5).

a Paired *t*-test.

**Table 2:** Structural parameters in the fibula of 19 week old female C57BL/6 mice treated with 6-weeks of vehicle or iPTH (1–34) and 2-weeks of mechanical loading with different peak loads producing a similar, lower level of strain

Peak load	12.0 N	15.8 N	*p* value^a^		
Dose of PTH (1–34)	0 μg/kg/day	80 μg/kg/day	Loading	iPTH (1–34)	Interaction
*Proximal site*					
Cortical bone volume (mm^3^)					
Left control	0.058 ± 0.004	0.088 ± 0.003	< 0.01	< 0.01	< 0.01
Right loaded	0.079 ± 0.009	0.141 ± 0.004			

*Middle site*					
Cortical bone volume (mm^3^)					
Left control	0.047 ± 0.002	0.065 ± 0.002	< 0.01	< 0.01	0.05
Right loaded	0.056 ± 0.005	0.087 ± 0.002			
Periosteally-enclosed volume (mm^3^)					
Left control	0.051 ± 0.002	0.075 ± 0.002	< 0.01	< 0.01	0.08
Right loaded	0.057 ± 0.005	0.094 ± 0.004			
Medullary volume (mm^3^)					
Left control	0.0046 ± 0.0006	0.0103 ± 0.0008	< 0.01	< 0.01	0.82
Right loaded	0.0016 ± 0.0004	0.0069 ± 0.0014			

*Distal site*					
Cortical bone volume (mm^3^)					
Left control	0.041 ± 0.002	0.062 ± 0.004	0.28	< 0.01	0.38
Right loaded	0.041 ± 0.001	0.067 ± 0.003			

Mean ± S.E. (*n* = 5).

a Two-way ANOVA.

**Table 3:** Numbers of sclerostin-positive and total osteocytes in the fibula of 19 week old female C57BL/6 mice that received 2-consecutive days of mechanical loading with a peak load of 13.5 N

	Left control	Right loaded	*p* value^a^
*Proximal site*			
Number of sclerostin-positive osteocytes	100 ± 2	31 ± 2	< 0.01
Number of total osteocytes	135 ± 2	135 ± 3	0.83

*Middle site*			
Number of sclerostin-positive osteocytes	74 ± 3	31 ± 2	< 0.01
Number of total osteocytes	91 ± 3	94 ± 2	0.52

Mean ± S.E. (*n* = 6).

a Paired *t*-test.

## References

[1] Ehrlich P.J., Lanyon L.E. (2002). Mechanical strain and bone cell function: a review. Osteoporos. Int..

[2] O'Connor J.A., Lanyon L.E., MacFie H. (1982). The influence of strain rate on adaptive bone remodelling. J. Biomech..

[3] Lanyon L.E., Rubin C.T. (1984). Static vs dynamic loads as an influence on bone remodelling. J. Biomech..

[4] Pead M.J., Suswillo R., Skerry T.M., Vedi S., Lanyon L.E. (1988). Increased 3H-uridine levels in osteocytes following a single short period of dynamic bone loading *in vivo*. Calcif. Tissue Int..

[5] Turner C.H., Akhter M.P., Raab D.M., Kimmel D.B., Recker R.R. (1991). A noninvasive, *in vivo* model for studying strain adaptive bone modeling. Bone.

[6] Chow J.W., Jagger C.J., Chambers T.J. (1993). Characterization of osteogenic response to mechanical stimulation in cancellous bone of rat caudal vertebrae. Am. J. Physiol..

[7] Torrance A.G., Mosley J.R., Suswillo R.F., Lanyon L.E. (1994). Noninvasive loading of the rat ulna *in vivo* induces a strain-related modeling response uncomplicated by trauma or periostal pressure. Calcif. Tissue Int..

[8] Akhter M.P., Cullen D.M., Pedersen E.A., Kimmel D.B., Recker R.R. (1998). Bone response to *in vivo* mechanical loading in two breeds of mice. Calcif. Tissue Int..

[9] Lee K.C., Maxwell A., Lanyon L.E. (2002). Validation of a technique for studying functional adaptation of the mouse ulna in response to mechanical loading. Bone.

[10] Gross T.S., Srinivasan S., Liu C.C., Clemens T.L., Bain S.D. (2002). Noninvasive loading of the murine tibia: an *in vivo* model for the study of mechanotransduction. J. Bone Miner. Res..

[11] De Souza R.L., Matsuura M., Eckstein F., Rawlinson S.C., Lanyon L.E., Pitsillides A.A. (2005). Non-invasive axial loading of mouse tibiae increases cortical bone formation and modifies trabecular organization: a new model to study cortical and cancellous compartments in a single loaded element. Bone.

[12] Fritton J.C., Myers E.R., Wright T.M., van der Meulen M.C. (2005). Loading induces site-specific increases in mineral content assessed by microcomputed tomography of the mouse tibia. Bone.

[13] Lee K., Jessop H., Suswillo R., Zaman G., Lanyon L. (2003). Endocrinology: bone adaptation requires oestrogen receptor-alpha. Nature.

[14] Lee K.C., Jessop H., Suswillo R., Zaman G., Lanyon L.E. (2004). The adaptive response of bone to mechanical loading in female transgenic mice is deficient in the absence of oestrogen receptor-alpha and -beta. J. Endocrinol..

[15] Sawakami K., Robling A.G., Ai M., Pitner N.D., Liu D., Warden S.J. (2006). The Wnt co-receptor LRP5 is essential for skeletal mechanotransduction but not for the anabolic bone response to parathyroid hormone treatment. J. Biol. Chem..

[16] Robling A.G., Niziolek P.J., Baldridge L.A., Condon K.W., Allen M.R., Alam I. (2008). Mechanical stimulation of bone *in vivo* reduces osteocyte expression of Sost/sclerostin. J. Biol. Chem..

[17] Sugiyama T., Saxon L.K., Zaman G., Moustafa A., Sunters A., Price J.S. (2008). Mechanical loading enhances the anabolic effects of intermittent parathyroid hormone (1–34) on trabecular and cortical bone in mice. Bone.

[18] de Souza R.L., Pitsillides A.A., Lanyon L.E., Skerry T.M., Chenu C. (2005). Sympathetic nervous system does not mediate the load-induced cortical new bone formation. J. Bone Miner. Res..

[19] Armstrong V.J., Muzylak M., Sunters A., Zaman G., Saxon L.K., Price J.S. (2007). Wnt/beta-catenin signaling is a component of osteoblastic bone cell early responses to load-bearing and requires estrogen receptor alpha. J. Biol. Chem..

[20] Marenzana M., De Souza R.L., Chenu C. (2007). Blockade of beta-adrenergic signaling does not influence the bone mechano-adaptive response in mice. Bone.

[21] Fritton J.C., Myers E.R., Wright T.M., van der Meulen M.C. (2008). Bone mass is preserved and cancellous architecture altered due to cyclic loading of the mouse tibia after orchidectomy. J. Bone Miner. Res..

[22] Saxon L.K., Lanyon L.E. (2008). Assessment of the *in vivo* adaptive response to mechanical loading. Methods Mol. Biol..

[23] Baron R., Vignery A., Neff L., Silverglate A., Santa Maria A., Recker RR (1983). Processing of undecalcified bone specimens for bone histomorphometry. Bone histomorphometry: techniques and interpretation.

[24] Zaman G., Jessop H.L., Muzylak M., De Souza R.L., Pitsillides A.A., Price J.S. (2006). Osteocytes use estrogen receptor alpha to respond to strain but their ERalpha content is regulated by estrogen. J. Bone Miner. Res..

[25] Keller H., Kneissel M. (2005). SOST is a target gene for PTH in bone. Bone.

[26] Vatsa A., Breuls R.G., Semeins C.M., Salmon P.L., Smit T.H., Klein-Nulend J. (2008). Osteocyte morphology in fibula and calvaria — is there a role for mechanosensing?. Bone.

[27] Robinson J.A., Chatterjee-Kishore M., Yaworsky P.J., Cullen D.M., Zhao W., Li C. (2006). Wnt/beta-catenin signaling is a normal physiological response to mechanical loading in bone. J. Biol. Chem..

[28] Srinivasan S., Weimer D.A., Agans S.C., Bain S.D., Gross T.S. (2002). Low-magnitude mechanical loading becomes osteogenic when rest is inserted between each load cycle. J. Bone Miner. Res..

[29] Hankenson K.D., Ausk B.J., Bain S.D., Bornstein P., Gross T.S., Srinivasan S. (2006). Mice lacking thrombospondin 2 show an atypical pattern of endocortical and periosteal bone formation in response to mechanical loading. Bone.

[30] Sample S.J., Behan M., Smith L., Oldenhoff W.E., Markel M.D., Kalscheur V.L. (2008). Functional adaptation to loading of a single bone is neuronally regulated and involves multiple bones. J. Bone Miner. Res..

